# Oxygen-carrying nanoemulsions and respiratory hyperoxia eliminate tumor hypoxia–induced immunosuppression

**DOI:** 10.1172/jci.insight.174675

**Published:** 2025-03-24

**Authors:** Katarina Halpin-Veszeleiova, Michael P. Mallouh, Lucy M. Williamson, Ashley C. Apro, Nuria R. Botticello-Romero, Camille Bahr, Maureen Shin, Kelly M. Ward, Laura Rosenberg, Vladimir B. Ritov, Michail V. Sitkovsky, Edwin K. Jackson, Bruce D. Spiess, Stephen M. Hatfield

**Affiliations:** 1 Department of Pharmaceutical Sciences, New England Inflammation and Tissue Protection Institute, Bouve College of Health Sciences, Northeastern University, Boston, Massachusetts, USA.; 2 Department of Surgery, University of Massachusetts Chan Medical School, Worcester, Massachusetts, USA.; 3 Department of Pharmacology and Chemical Biology, University of Pittsburgh School of Medicine, Pittsburgh, Pennsylvania, USA.; 4 Department of Anesthesiology, College of Medicine, University of Florida, Gainesville, Florida, USA.; 5 Department of Chemical Engineering, College of Engineering, Northeastern University, Boston, Massachusetts, USA.

**Keywords:** Immunology, Oncology, Cancer, Cancer immunotherapy, Hypoxia

## Abstract

Hypoxia/hypoxia-inducible factor 1α–driven immunosuppressive transcription and cAMP-elevating signaling through A2A adenosine receptors (A2ARs) represent a major tumor-protecting pathway that enables immune evasion. Recent promising clinical outcomes due to the blockade of the adenosine-generating enzyme CD73 and A2AR in patients refractory to all other therapies have confirmed the importance of targeting hypoxia-adenosinergic signaling. We report a feasible approach to target the upstream stage of hypoxia-adenosinergic immunosuppression using an oxygen-carrying nanoemulsion (perfluorocarbon blood substitute). We show that oxygenation agent therapy (a) eliminates tumor hypoxia, (b) improves efficacy of endogenously developed and adoptively transferred T cells, and thereby (c) promotes regression of tumors in different anatomical locations. We show that both T cells and NK cells avoid hypoxic tumor areas and that reversal of hypoxia by oxygenation agent therapy increases intratumoral infiltration of activated T cells and NK cells due to reprogramming of the tumor microenvironment (TME). Thus, repurposing oxygenation agents in combination with supplemental oxygen may improve current cancer immunotherapies by preventing hypoxia-adenosinergic suppression, promoting immune cell infiltration and enhancing effector responses. These data also suggest that pretreating patients with oxygenation agent therapy may reprogram the TME from immunosuppressive to immune-permissive prior to adoptive cell therapy, or other forms of immunotherapy.

## Abstract 2

Oxygen delivering nanoemulsions and respiratory hyperoxia address limitations of blood vessel-mediated tumor oxygenation and promote anti-tumor immune responses to enhance immunotherapy.

## Introduction

The discovery of immune checkpoints motivated preclinical and clinical studies that have led to novel immunotherapeutic treatment modalities (1–4). While immune checkpoint blockade (ICB) has afforded durable clinical responses, many patients relapse or fail to respond. In contrast with these immunological barriers, here we explored the targeting of hypoxia-adenosinergic biochemical negative regulators, which have been demonstrated in both preclinical studies and clinical observations to induce immunosuppression in the tumor microenvironment (TME).

**Figure 1:**
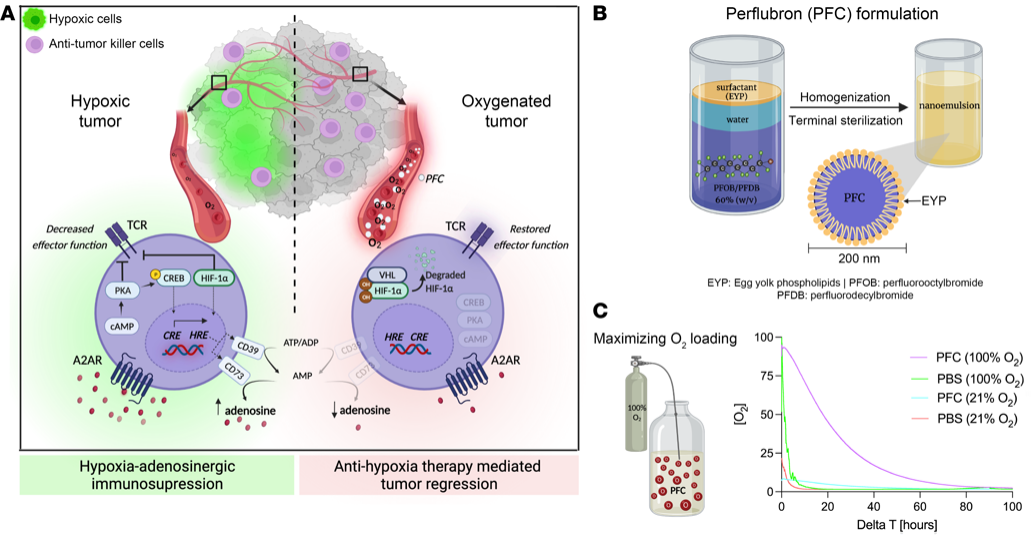
Exposure of PFC nanoemulsion to 100% O_2_ prior to in vivo administration maximizes levels of dissolved oxygen. (**A**) Schematic representation of the rationale to use PFC to reverse tumor hypoxia and target hypoxia-adenosinergic signaling. The hypoxia/HIF1A-driven increase in adenosine-generating ectoenzymes CD39 and CD73 leads to the accumulation of extracellular adenosine in the TME that triggers immunosuppressive A2AR → cAMP → PKA signaling. PFCs target the upstream stage of the hypoxia-adenosinergic axis of immunosuppression by increasing tumor oxygen tension and eliminating hypoxia/HIF1A- and A2AR-mediated signaling. Tumor oxygenation weakens all known stages of the hypoxia-adenosinergic signaling axis to prevent inhibition of antitumor cytotoxic killer cells and promote tumor regression (28, 29). HRE, hypoxia response element; CRE, cAMP response element; PKA, protein kinase A; CREB, cAMP response element binding protein; HIF1A, hypoxia-inducible factor 1α; VHL, von Hippel-Lindau; PFC, perfluorocarbon; TCR, T cell receptor. Created with BioRender.com. (**B**) Illustration depicting Perflubron, a PFC-based nanoemulsion consisting of 60% (w/v) perfluorooctylbromide (PFOB) and perfluorodecylbromide (PFDB) in water stabilized with egg yolk phospholipids (EYPs). After homogenization, the nanoemulsion consists of particles that are less than 200 nm in size. The amount of dissolved oxygen (O_2_) in Perflubron is proportional to the partial pressure of O_2_ in the environment. (**C**) Left: Illustration depicting the strategy to maximize oxygen-carrying capacity of Perflubron by saturation with 100% O_2_ for 7 minutes. Unsaturated samples were maintained at 21% O_2_. Right: One mL of unsaturated (21% O_2_) or saturated (100% O_2_) PFC or PBS as control was placed into an H35 HEPA Hypoxystation (Don-Whitley) maintained at 1% O_2_, 5% CO_2_, and 37°C. Dissolved oxygen concentration was measured continuously by the Presens oxygen monitoring system and Presens in vitro oxygen probes.

Hypoxia-adenosinergic signaling emerged as a potent immunomodulating mechanism that governs the duration and direction of antipathogen and antitumor immune responses. It was established (5–7) and confirmed (8–23) that the major signaling components that drive this immunosuppressive axis are G_s_-protein–coupled A2A adenosine receptors (A2ARs) that trigger the accumulation of cAMP and subsequent activation of downstream PKA-mediated signaling events (24). While this biochemical pathway inhibits effector functions of many immune cell subtypes, it is most well documented in T cells, where transmembrane signaling from A2ARs delivers an “off signal” to early and late events of TCR signaling as well as p-CREB–mediated transcriptional changes in genes with cAMP response elements (CREs) (Figure 1A) (25). In the context of the hypoxic TME, this hypoxia/hypoxia-inducible factor 1α–driven (HIF1A-driven) and adenosine-mediated immunosuppressive axis leads to the inhibition of the antitumor immune response via reduction of cytokine production, proliferation, and cytolytic capacity, among other suppressive mechanisms (5, 7, 26–33).

Hypoxia-adenosinergic immunoregulation is now established as a critical mechanism that protects cancerous tissues and drives resistance to cancer therapies. This was based on previous insights from genetic, biochemical, and pharmacological landmark studies (5, 7) demonstrating that hypoxia-adenosinergic signaling functions as a general mechanism to protect normal cells from excessive collateral damage by overactive antipathogen immune responses, but also protects cancerous cells. Hypoxia-adenosinergic immunosuppression was discovered in the context of inflammation, where damage to the endothelium and subsequent hypoxia increase the expression of adenosine-generating enzymes (e.g., CD39 and CD73) (10–13, 34–38), leading to the accumulation of extracellular adenosine that signals through cAMP-elevating A2AR and A2BR. However, it was soon demonstrated that this fundamental, tissue-protecting mechanism is hijacked by tumor cells in the hypoxic, adenosine-rich TME to suppress antitumor immune responses (7).

Of note, the hypoxia-adenosinergic biochemical barrier in the TME also promotes and strengthens immunological barriers by augmenting suppression by immune checkpoints. A2AR-mediated signaling has been shown to increase levels of immunological negative regulators, including LAG-3, IL-10, and T regulatory cells (Tregs), among others (15, 39). Additional lines of evidence also suggest that hypoxia/HIF1A signaling promotes expression of immunological barriers such as CTLA-4, LAG-3, PD-1, and TIM-3 (28, 40). Crosstalk between hypoxia/HIF1A and A2-adenosinergic signaling has been further confirmed in studies of cAMP-dependent PKA signaling, where phosphorylation of PKA was shown to recruit the transcriptional activity of HIF1A (41). Furthermore, the adenosine-generating ectoenzymes CD39 and CD73 are regulated by HIF1A and have been shown to promote tumor growth, invasiveness, and metastatic potential, resulting in poor clinical outcomes (8, 9, 16–20, 28, 29, 35, 36, 42).

The discovery of this tumor-protecting biochemical pathway advanced our understanding of pharmacological approaches to block the early stages (hypoxia/HIF1A) and late stages (adenosine/A2AR/cAMP) of this signaling axis. Preclinical evidence for this approach employing antihypoxia and anti-A2AR strategies has led to the justification of multiple clinical trials using such drugs against cancers that are refractory to all other therapies. Therefore, there is an acute need to improve outcomes of patients who are unresponsive to treatments such as ICB since these tumors are still protected by the hypoxia-adenosinergic immunosuppressive barrier.

Here, we provide preclinical evidence for the improvement of cancer immunotherapies by targeting hypoxia, the upstream stage of the hypoxia-adenosinergic axis, using an oxygenation agent, perfluorocarbon (PFC), in combination with respiratory hyperoxia (hereafter referred to as “oxygenation agent therapy”) (43). This study was guided by our previous work demonstrating that the weakening of tumor hypoxia following supplemental oxygenation (respiratory hyperoxia, 60%) targets all downstream stages of the hypoxia-adenosinergic immunosuppressive pathway by decreasing levels of HIF1A, extracellular adenosine, CD39, CD73, and A2AR/A2BR (Figure 1A) (28, 29). Since oxygenation of the TME interrupts both HIF1A and adenosine signaling pathways, this allowed us to provide the missing evidence to directly connect hypoxia/HIF1A (upstream) as governing the downstream CD39/CD73 → [adenosine] → A2AR/A2BR → intracellular cAMP → PKA → CRE → CREB pathway. These studies also demonstrated that by targeting this pathway, the reversal of tumor hypoxia could reprogram the immunosuppressive metabolome, proteome, and cytokinome profile to improve antitumor responses (28, 29).

Our approach to combine oxygenation agents with respiratory hyperoxia was motivated by our goal to extend the penetration of oxygen into depths of solid tumors to better eliminate tumor hypoxia, especially in areas where damaged vasculature does not enable the diffusion of oxygen during respiratory hyperoxia. Thus, in this study we sought to improve the delivery of oxygen further by using oxygenation agents that are readily available and shown to be safe in humans.

PFCs may address limitations of respiratory hyperoxia that may be ineffective in increasing oxygen levels in tumor areas that are not reached by healthy blood vessels, have marked vascular damage, or are in anatomical areas that may not be as oxygen-privileged as the lungs. PFCs overcome these impediments since they are much smaller in size compared with erythrocytes and oxygen is physically dissolved in the PFC nanoemulsion, rather than chemically bound as in hemoglobin (44–47). We suggest here what we believe is a conceptually novel immunologically motivated use of blood substitutes (e.g., oxygenation agents) to enhance oxygen delivery to the TME, thereby weakening hypoxia-adenosinergic immunosuppression and improving cancer immunotherapies (43). This approach differs from previous strategies utilizing PFCs to enhance the therapeutic benefits of radiotherapy, phototherapy, and chemotherapy (45, 48, 49). While oxygenation agents were first considered to be combined with immunotherapies by Ohta and Sitkovsky (50), this was never tested to the best of our knowledge. In this report, we present our findings on the synergy between 2 antihypoxia treatments — respiratory hyperoxia and oxygen-carrying nanoemulsions.

These studies may resurrect an entire industry of blood substitutes that were developed previously for other indications, including stroke, military applications, organ preservation, and as blood substitutes during the AIDS epidemic (51–53). Our unique motivation to use oxygen-carrying nanoemulsions to eliminate tumor hypoxia as the upstream stage of the hypoxia-adenosinergic immunosuppressive axis provides rationale to repurpose these drugs to improve cancer immunotherapy. Importantly, PFC nanoemulsions have previously been tested in over 3,000 patients and shown to be safe for other indications (e.g., stroke, ischemia, cardiac surgery, decompression illness, etc.) (12, 13, 47, 51, 53). In addition, over 1,100 studies have been completed using PFCs and external safety studies have been conducted (4, 8–11). Support for such an approach is also provided by preclinical and clinical studies by Curran’s group demonstrating improved therapeutic efficacy utilizing a related strategy with a hypoxia-targeting prodrug, evofosfamide (23, 54).

## Results

### Optimizing oxygen delivery by PFC nanoemulsions to target the upstream stage of hypoxia-adenosinergic signaling axis.

Low levels of O_2_ (PO_2_ < 10 mmHg ≈ 1.4% O_2_) in the TME are associated with much lower disease-free survival in cancer patients when compared with patients with higher mean intratumoral PaO_2_ (55–57). We have proposed that the hypoxic TME is a major obstacle preventing success of cancer immunotherapies (Figure 1A), including adoptive cell therapy (ACT) and ICB. While recent attention has focused on immunological barriers (ICB) in the TME, we have provided justification for the targeting of biochemical barriers (e.g., hypoxia-adenosinergic suppression) that hinder infiltration, proliferation, and effector functions of antitumor immune cells (23, 24, 28, 29). Our previous work has shown that hypoxic and adenosine-rich tumors are protected from attack by antitumor immune cells (28, 29, 33).

To address limitations of oxygen delivery via erythrocytes, we investigated the use of the PFC-based oxygenation agent, Perflubron, as an approach to target tumor hypoxia, the upstream stage of the hypoxia-adenosinergic immunosuppressive signaling axis. We hypothesized that the small size (<0.2 μm) of these particles will allow better penetration in narrow microvascular channels of solid tumors (58). Figure 1B depicts the composition of Perflubron (PFC), a mixture of 60% (w/v) perfluorooctyl bromide (PFOB) and perfluorododecyl bromide (PFDB) in water stabilized with egg yolk phospholipids (EYPs) (47). Here, we exploited the oxygen solubility of PFC, which is directly proportional to the oxygen partial pressure in the surrounding environment (Henry’s law) (58). Perflubron “off-the-shelf” has a concentration of dissolved O_2_ similar to atmospheric air (~21% O_2_). We speculated that oxygenating Perflubron by exposure to 100% O_2_ would maximize the amount of dissolved oxygen in the PFC nanoemulsion prior to in vivo administration, thereby increasing oxygen delivering capacity (Figure 1C).

As shown in Figure 1C, we exposed Perflubron to 100% O_2_ for 7 minutes and compared the oxygen dissolving and release capacity of oxygenated Perflubron to the “off-the-shelf” preparation of Perflubron. To mimic the hypoxic TME, these in vitro studies were conducted in an H35 Hypoxystation (Don-Whitley) in 1% O_2_/5% CO_2_ using a PreSens oxygen monitoring system to measure levels of dissolved oxygen. Figure 1C demonstrates that the oxygen-carrying capacity of oxygenated Perflubron was increased and the oxygen release profile was prolonged compared with control, non-oxygenated Perflubron. These in vitro studies suggest a potential benefit of exposing Perflubron to high oxygen prior to administration to enhance oxygen-carrying capacity, which might also be achieved in vivo by combining with respiratory hyperoxia. The data from Figure 1 provided a methodological rationale for (a) oxygenating Perflubron prior to in vivo administration for maximized initial O_2_ loading and (b) combination therapy with respiratory hyperoxia during Perflubron administration (oxygenation agent therapy) to achieve higher fractions of inspired oxygen (F_i_O_2_) and maximize the amount of dissolved O_2_ in Perflubron after in vivo administration.

### Therapeutic targeting of hypoxia-governed immunosuppression via oxygenation agent therapy.

To alleviate the inhibition of antitumor immunity in hypoxic tumors, we tested whether oxygenation agent therapy could reverse tumor hypoxia and reprogram the TME (28). For these studies, mice with established MCA205 fibrosarcoma intradermal tumors were given daily administration of Perflubron for 72 hours with and without respiratory hyperoxia (60% O_2_). Respiratory hyperoxia using 60% O_2_ is established as a safe protocol in long-term studies both preclinically in mice and clinically in humans (28, 29, 59, 60).

**Figure 2:**
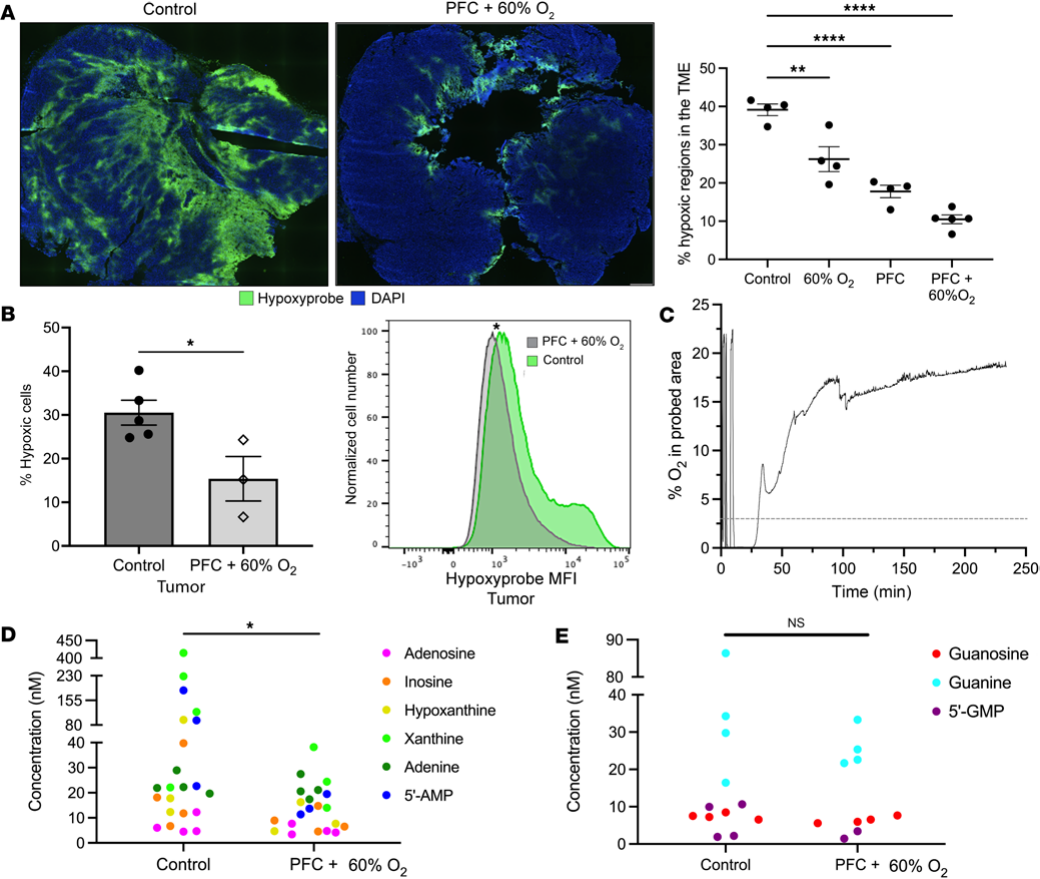
Oxygenation agent therapy eliminates intratumoral hypoxia and reduces levels of downstream metabolites of extracellular adenosine in the TME. Mice with 8-day-established MCA205 intradermal tumors were treated with or without i.v. administration of 15 mL/kg oxygenated Perflubron (PFC) for 72 hours with continuous breathing of 60% O_2_ (respiratory hyperoxia) or 21% O_2_ (normoxia). Intratumoral hypoxia was measured by immunofluorescence (**A**) and flow cytometry (**B**) using the hypoxia marker Hypoxyprobe-1 injected i.p. (80 mg/kg) 1 hour prior to sacrifice. (**A**) Left: Representative fluorescence images of hypoxia levels (green) in the isolated tumors from control versus mice treated with PFC plus 60% O_2_. Right: Quantification of intratumoral hypoxia using CellSens software (Olympus) in all experimental groups. *P* values were calculated using 1-way ANOVA with Tukey’s post hoc test for multiple comparisons. *P < 0.05; **P < 0.005; ****P < 0.0001. Data presented as mean ± SEM, *n* ≥ 4. (**B**) Left*:* Tumors isolated from mice treated with oxygenation agent therapy (white bars) had substantially fewer hypoxic cells compared with control (gray bars) as measured by flow cytometric analysis of Hypoxypobe staining (*P* = 0.02). Right*:* Increased mean fluorescence intensity (MFI) of Hypoxyprobe staining on cells isolated from tumors from control mice (green) and mice treated with oxygenation agent therapy (gray). *P* values were calculated using 1-way ANOVA with Tukey’s post hoc test for multiple comparisons. *P < 0.05, *n* ≥ 4. (**C**) Real-time measurement of increased intratumoral oxygen concentration in intradermal tumors after Perflubron infusion measured by Presens oxygen monitoring system and Presens in vivo probes. After stabilization of the oxygen probe in the hypoxic tumor region (0.02% O_2_) in an anesthetized mouse, Perflubron was infused i.v. (15 mL/kg) at minute 12. Oxygen concentration was recorded up to 229.5 minutes. The oxygen concentration curve at the probed tumor area is shown as a function of time before and after Perflubron administration (arrow indicates Perflubron administration and dotted line indicates oxygen concentration where HIF1A is considered destabilized). Demonstration of increased intratumoral oxygen tension by Perflubron administration was repeated in 3 independent experiments. (**D** and **E**) Mice bearing intradermal CT26 tumors were treated with and without oxygenation agent therapy (PFC + 60% O_2_) for 24 hours (*n* = 4 mice per group). PFC or PBS was administered i.v. at 0 and 24 hours, mice were anesthetized, and equilibrium microdialysis probes were inserted into the center of tumors to collect microdialysate. Adenosine (**D**) and guanosine (**E**) purine metabolites were measured by HPLC–tandem quadrupole mass spectrometry. *P < 0.05; NS, not significant by unpaired *t* test.

In Figure 2A, representative immunofluorescence images and quantification of Hypoxyprobe staining of tumor sections demonstrate that oxygenation agent therapy reversed hypoxia in intradermal tumors. While a decrease in hypoxic regions was observed in mice receiving Perflubron alone, the nearly complete elimination of hypoxia was seen in mice treated with Perflubron in combination with respiratory hyperoxia (60% O_2_) (Figure 2A and Supplemental Figure 1A; supplemental material available online with this article; https://doi.org/10.1172/jci.insight.174675DS1). These data were confirmed by flow cytometry assays demonstrating that the percentage and mean fluorescence intensity (MFI) of hypoxic tumor cells was greatly decreased in mice treated with Perflubron in combination with respiratory hyperoxia, with statistical significance observed (*P* = 0.02, Figure 2B; *P* < 0.05, Supplemental Figure 1B).

We then asked whether we could measure the increased oxygen levels in the tumor following Perflubron administration in real time. To this end, we employed the PreSens oxygen monitoring system to measure oxygen levels in anesthetized mice bearing intradermal tumors. After locating hypoxic tumor areas (<1% O_2_) using an in vivo Presens oxygen probe, we infused Perflubron and continuously monitored oxygen tension. Twelve minutes after administration of Perflubron, the intratumoral oxygen levels increased to approximately 15% O_2_ (pO_2_ ≈ 107 mmHg) and was sustained for the duration of the assay (~250 minutes). Figure 2C shows the real-time measurements of the increase in oxygen concentration over the 4-hour time period.

Since our goal is to decrease the hypoxia/HIF1A-mediated triggering of immunosuppressive downstream events, it is important to emphasize that 10–15 mmHg (1.5%–2% O_2_) is considered the 50% inhibitory concentration of the catalytic subunit of HIF1A (57, 61). Therefore, Figures 1 and 2 represent mechanistic biochemical justification for using our approach in vivo since, according to published reports (62), at the partial pressure of oxygen achieved by oxygenation agent therapy (PO_2_ ≈ 107 mmHg), HIF1A is destabilized (62). According to clinical data, such an increase in oxygen concentration has dramatic consequences in the responses and outcomes of cancer patients. Of note, previous work by Dewhirst and colleagues showed that patients with intratumoral PaO_2_ greater than 11 mmHg have over 50% higher chance of disease-free survival (56).

Additional assays also demonstrated that oxygenation agent therapy not only affected levels of tumor hypoxia, but also the hypoxic exposure of tumor-infiltrating immune cells. We show in Supplemental Figure 2 that oxygenation agent therapy substantially reduced the hypoxic exposure of antitumor CD4^+^ T cells (Supplemental Figure 2A), CD8^+^ T cells (Supplemental Figure 2B), and NK cells (Supplemental Figure 2C). We and others have previously established that hypoxia inhibits many effector functions of T cells and NK cells, including cytokine production, proliferation, and cytotoxicity (1, 18–21). Supplemental Figure 2D also demonstrates that immunosuppressive Tregs have less hypoxic exposure following oxygenation agent therapy. Implications of this finding may be important since hypoxia promotes Treg-mediated immunosuppression by triggering hypoxia-adenosinergic transcription and producing TGF-β (*TGFB*), IL-10, and other immunoregulatory molecules (22) that inhibit antitumor T cells and NK cells.

We previously showed that reversal of hypoxia-HIF1A by systemic oxygenation reduces expression of adenosine-generating enzymes CD39 and CD73, as well as extracellular adenosine itself. Therefore, we sought to expand the coverage of the purinome to include analysis of major adenosine metabolites (e.g., adenosine, inosine, xanthine, hypoxanthine, adenine, 5′-AMP). Mice bearing CT26 intradermal tumors were treated with and without oxygenation agent therapy (PFC + 60% O_2_) for 24 hours and equilibrium microdialysis probes were inserted into the center of tumors. Purine levels were measured by reversed-phase liquid chromatography–tandem mass spectrometry. Figure 2D demonstrates that the sum total of all adenosine metabolites was significantly reduced (*P* = 0.02) following oxygenation agent therapy compared with control. In an important internal control, guanosine metabolites were not significantly changed following oxygenation agent therapy (Figure 2E). Analysis of the purinome may represent a new methodological and technical advance, since adenosine is rapidly degraded in vivo into these metabolites. Moreover, recent studies have shown that some of these purine metabolites can be immunosuppressive on their own and alter signaling through adenosine receptors (63, 64).

### Oxygenation agent therapy reprograms the TME to promote antitumor immune cell infiltration.

**Figure 3:**
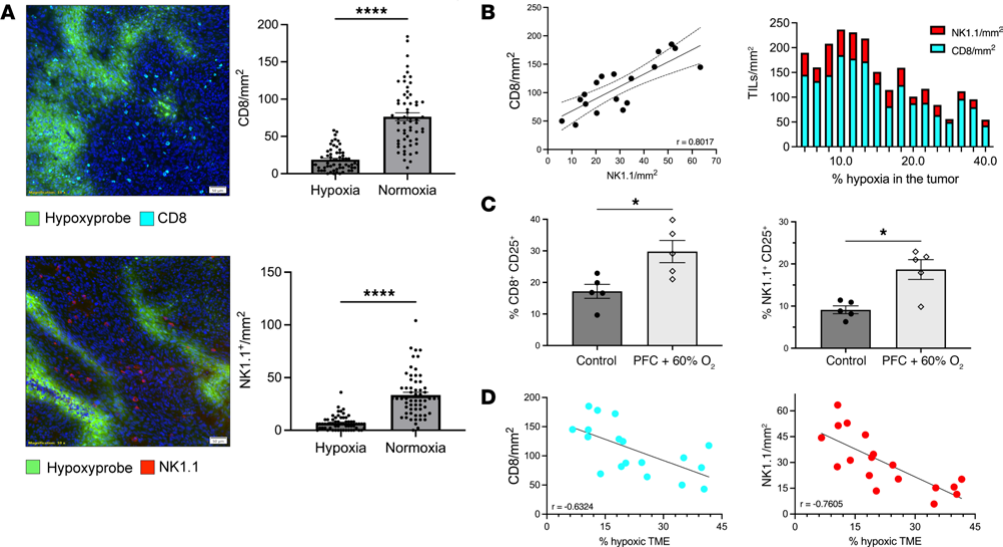
Oxygenation agent therapy reprograms the TME toward immune-permission. (**A**) Antitumor T cells and NK cells avoid hypoxic regions of solid tumors. MCA205 intradermal tumors were isolated and 5-μm frozen tissue sections were prepared. Fixed sections were fluorescently labeled with anti-Hypoxyprobe, anti-CD8, and anti-NK1.1 antibodies to determine infiltration of immune cells into the hypoxic (green) or nonhypoxic regions (dark blue, DAPI). Left panels: Representative fluorescence images demonstrating that CD8^+^ T cells (top, light blue) and NK cells (bottom, red) avoid hypoxic regions (green). Right panels: Quantification of CD8^+^ T cell (top) and NK cell (bottom) infiltration into hypoxic versus nonhypoxic areas. A total of 60 different regions from the TME across 15 tumors were analyzed using CellSens software (Olympus) and results are shown in the bar graphs (right). ****P < 0.0001 by unpaired *t* test. Data presented as mean ± SEM. Scale bar: 50 μm (original magnification, ×10). (**B**–**D**) C57BL/6 mice with 8-day-established MCA205 intradermal tumors received daily administration (i.v., 15 mL/kg) of oxygenated Perflubron for 72 hours while breathing 60% O_2_ (respiratory hyperoxia) or 21% O_2_ (normoxia). Mice were injected i.p. with Hypoxyprobe-1 (80 mg/kg) 1 hour before sacrifice to determine levels of intratumoral hypoxia. After sacrifice, tumors were isolated and divided for analysis via immunofluorescence or flow cytometry. (**B**) Left: Quantitative analysis using CellSens software (Olympus) of 5-μm-thick frozen tumor sections correlating the number of infiltrating NK cells and CD8^+^ cells per tumor. Each dot represents 1 tumor (*n* = 18). Dotted line represents 95% CI. Right: Bar graph depicting the relationship between NK cells (red) and CD8^+^ cells (blue) and levels of intratumoral hypoxia. Each bar represents 1 tumor and corresponding numbers for infiltration and hypoxic regions (*n* = 18). TILs, tumor-infiltrating lymphocytes. (**C**) Quantitation of the numbers CD8^+^CD25^+^ (left) and NK^+^CD25^+^ (right) cells from tumors from mice treated with oxygenation agent therapy versus control. *P* values were calculated using 1-way ANOVA with Tukey’s post hoc test for multiple comparisons. *P < 0.05. Data are presented as mean ± SEM, *n* = 5. (**D**) Pearson’s correlation analyses between the percentage of hypoxic regions in the TME and the number of infiltrating CD8^+^ T cells (left) or NK cells (right). Each dot represents 1 tumor (*n* = 18).

Since we proposed that tumor hypoxia is a powerful immunosuppressive barrier, we then examined the infiltration of antitumor immune cells into normoxic versus hypoxic regions of tumors. Data from Figure 3A extend previous observations by demonstrating that both antitumor CD8^+^ T cells and NK cells avoid hypoxic regions of intradermal tumors as measured by the biochemical hypoxia marker, Hypoxyprobe (23, 28). Significantly fewer CD8^+^ T cells (*P* < 0.0001, Figure 3A) and NK cells (*P* < 0.05, Figure 3B) were observed in hypoxic tumor areas (<1% O_2_) in all of the intradermal tumors assayed.

The absence of CD8^+^ T cells and NK cells in hypoxic regions led us to investigate whether reprogramming the TME by reversing tumor hypoxia (Figure 2) (29) (28) might affect recruitment of endogenous antitumor immune cells. Using fluorescence microscopy, we quantified the numbers of CD8^+^ T cells, NK cells, and hypoxia from intradermal tumors. Mice were treated with daily administration of Perflubron and respiratory hyperoxia (60% O_2_) for 72 hours or kept at atmospheric oxygen levels as a control. We first evaluated the relationship between infiltrating CD8^+^ T cells and NK cells regardless of hypoxic levels in the TME. Figure 3B shows a strong positive correlation between the number of infiltrating T cells and NK cells into tumors (*r* = 0.8), supporting previous observations that NK cells may recruit and orchestrate activities of CD8^+^ T cells (28). We then examined the number of T cell and NK cell infiltrates in the context of tumor hypoxia. Figure 3B demonstrates that tumors with a lower percentage of hypoxic regions had higher levels of infiltrating T cells and NK cells. These observations are in agreement with findings from Figure 3A. Taken together, these data suggest that the mechanisms that attract or inhibit T cell or NK cell infiltration are strongly affected by tumor hypoxia. This is supported by our previous findings that the reversal of hypoxia greatly increased the expression of proinflammatory cytokines and chemokines in the TME (28).

Next, we asked whether reprogramming of the TME by oxygenation agent therapy would increase the recruitment and activation of antitumor immune cells. Figure 3C and Supplemental Figure 3 show that oxygenation agent therapy resulted in significantly increased numbers of intratumoral CD8^+^CD25^+^ and NK1.1^+^CD25^+^ cells. We then examined the correlation between hypoxia and CD8^+^ T cells or NK cells. Figure 3D demonstrates a strong negative correlation between CD8^+^ T cell infiltration and hypoxia (*r* = –0.6324), and an even stronger negative correlation between NK cells and hypoxic levels (*r* = –0.7605). These data suggest that NK cells may be more sensitive to immunosuppressive hypoxic signaling. This supports previous demonstrations of the high susceptibility of NK cells to adenosine-A2AR–mediated suppression, which is prominent in hypoxic conditions (28, 65, 66).

### Oxygenation agent therapy induces tumor regression and improves survival.

**Figure 4:**
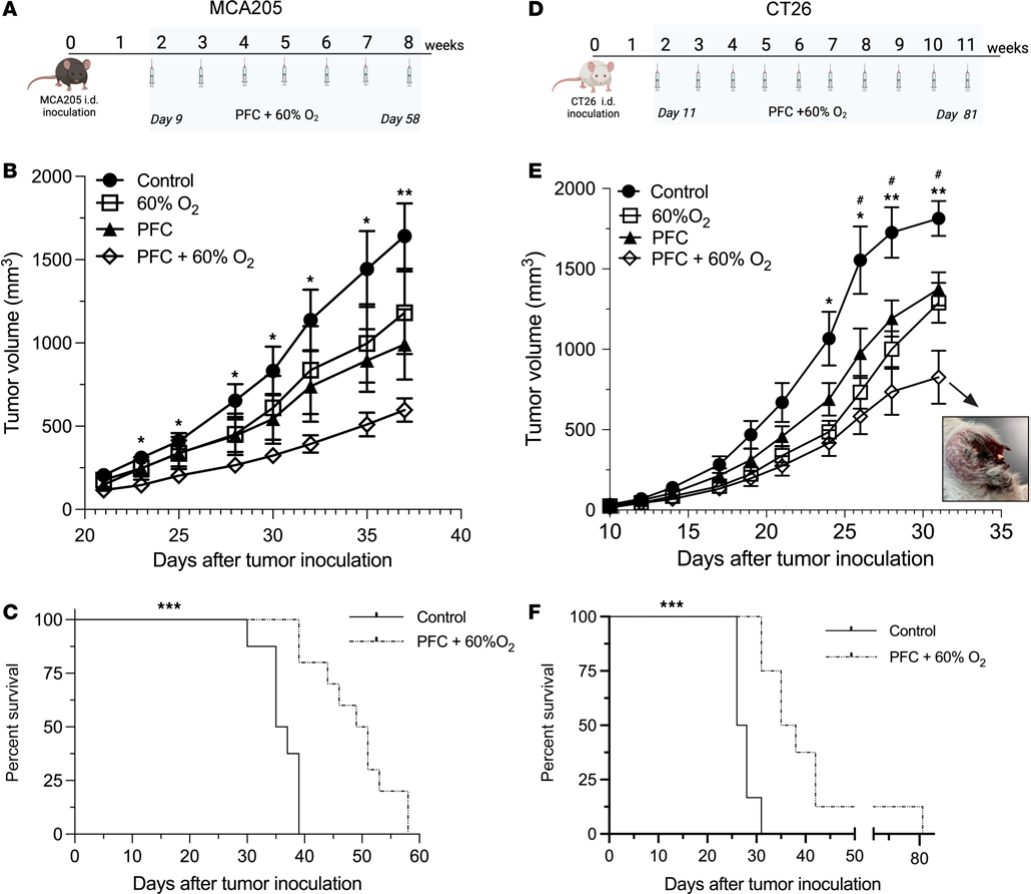
Oxygenation agent therapy induces tumor regression and improves survival. C57BL/6 and BALB/c mice were injected i.d. with MCA205 fibrosarcoma or CT26 colon carcinoma and treated with or without Perflubron (PFC) and respiratory hyperoxia (60% O_2_). (**A**) Schematic illustration of the experimental design and treatment regimen in the MCA205 intradermal model. C57BL/6 mice with 9-day-established MCA205 fibrosarcoma intradermal tumors were housed in 60% O_2_ chambers or maintained at 21% O_2_ (normoxia) and treated with or without oxygenated Perflubron (15 mL/kg) 3 times per week until study completion. Created with BioRender. (**B**) Oxygenation agent therapy (PFC + 60% O_2_) induces the strongest tumor regression compared with control or either treatment alone. Tumors were measured 3 times per week using Vernier calipers and tumor volume was calculated by the following formula: π/6 × *H* × *W* × *L*. *P < 0.05, **P < 0.005. Data presented as mean ± SEM, *n* ≥ 7. (**C**) Survival curves for MCA205 tumor–bearing mice treated with oxygenation agent therapy (PFC + 60% O_2_) versus control (*P* < 0.001, *n* ≥ 9). (**D**) Schematic illustration of the experimental design and treatment regimen in the CT26 intradermal tumor model. BALB/c mice with 11-day-established CT26 intradermal tumors were housed in 60% O_2_ chambers or maintained at 21% O_2_ with or without oxygenated Perflubron (10 mL/kg) 3 times per week until study completion. Created with BioRender. (**E**) Oxygenation agent therapy (PFC + 60% O_2_) induces the strongest tumor regression compared with control or either treatment alone. Evaluation of tumor growth kinetics and analysis is identical to **B**. *P < 0.05, **P < 0.005 for control versus PFC + 60% O_2_; ^#^*P* < 0.05 for control versus 60% O_2_. Data are presented as mean ± SEM, *n* ≥ 6. Inset: Representative image of ulceration, necrosis, and large holes observed in tumors from mice treated with oxygenation agent therapy. (**F**) Survival curves for CT26 tumor–bearing mice treated with oxygenation agent therapy (PFC + 60% O_2_) versus control. ***P < 0.001, *n* ≥ 6. Statistical significance was assessed using 2-way ANOVA with Tukey’s multiple-comparison test (**B**, **C**, **E**, and **F**).

After demonstrating that oxygenation agent therapy eliminates tumor hypoxia and increases the infiltration of tumor-reactive lymphocytes, we tested whether this approach is capable of inducing tumor regression. Figure 4, A and B show that while Perflubron alone and respiratory hyperoxia (60% O_2_) alone resulted in a slight tumor growth delay, oxygenation agent therapy (3 Perflubron injections/week + 60% O_2_) promoted further regression of 9-day-established intradermal MCA205 fibrosarcoma tumors that was statistically significant when compared with control mice (*P* < 0.05, Figure 4A). This potent tumor regression induced by oxygenation agent therapy was also confirmed in the CT26 colon carcinoma model in Figure 4, D and E (*P* < 0.05). These data extend previous findings of the immunological antitumor effects of respiratory hyperoxia in lung tumor models that are much more readily oxygenated by breathing 60% O_2_ alone (28, 29). Interestingly, we observed striking ulceration and necrosis in tumors from mice treated with oxygenation agent therapy, which may indicate morphological signs of tumor destruction by antitumor immune cells (Figure 4E, inset). This phenomenon was not observed in tumors from other groups. Additional mechanistic assays confirmed that these tumor-bearing mice treated with oxygenation agent therapy exhibited decreased intratumoral hypoxia at the study end point (Supplemental Figure 3). Figure 4, C and F demonstrate that oxygenation agent therapy also significantly improved the survival of intradermal tumor–bearing mice compared with untreated control mice in 2 separate tumor models (CT26 and MCA205). Taken together, these data suggest that oxygenation agent therapy eliminates tumor hypoxia, promotes infiltration of activated antitumor immune cells, and induces marked tumor regression, particularly in anatomical locations that are not as oxygen privileged as the lung.

### Oxygenation agent therapy improves outcomes of ACT.

The maximum therapeutic benefit of oxygenation agent therapy is likely to be achieved only when combined with other cancer immunotherapies, such as ACT (43). In this study, we suggest a feasible solution to one of the main impediments preventing therapeutic success of ACT in solid tumors — limited intratumoral infiltration/engraftment of T cells and suppression within the hostile TME. We, and others, have established that the hypoxic and adenosine-rich TME inhibits infiltration of endogenous and adoptively transferred antitumor killer cells and downregulates effector functions and cytolytic activity (7, 8, 16, 28, 29, 67, 68). While many current cancer immunotherapy protocols assume that patients have sufficient antitumor immune cells, we suggest that only ACT may be capable of delivering sufficiently high numbers to achieve clinical success (68). However, clinical outcomes of ACT in patients with solid tumors, even with chimeric antigen receptor (CAR) T cells, suggest that high numbers of transferred cells are insufficient due to inhibition by immunological and biochemical barriers in the TME.

**Figure 5:**
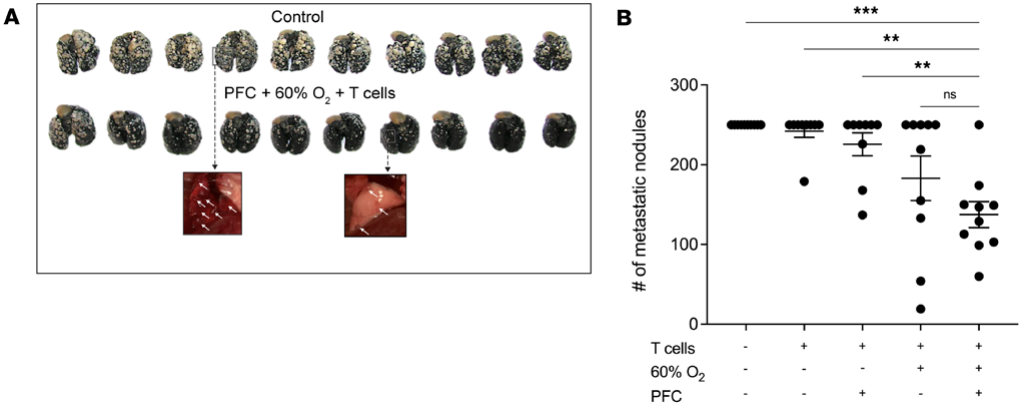
Oxygenation agent therapy improves efficacy of adoptive T cell therapy. Mice with 11-day-established pulmonary metastases received ACT of 10 × 10^6^ T cells. One day prior to ACT, mice underwent lymphodepletion with 100 mg/kg i.p. cyclophosphamide to mimic clinical protocols. Several hours prior to ACT, mice received i.v. administration of 10 mL/kg of PFC followed by 5 additional doses on days 12, 13, 14, 17, and 19. On day 11 (same day as ACT), mice were placed in a 60% O_2_ chamber to maximize PFC O_2_ transport or maintained at 21% O_2_ as control until assay completion on day 21. After termination of the study, mice were sacrificed, and lung tumors enumerated by counterstaining with India ink. (**A**) Images of tumor-bearing lungs from control versus PFC + 60% O_2_ + ACT (white arrows indicate metastatic nodules on the lungs prior to counterstaining). (**B**) Quantification of the number of metastatic nodules. Lungs with more than 250 tumors were denoted as such since this is the maximum number that can be counted reliably. *P* values were calculated using 1-way ANOVA with post hoc Tukey’s HSD. **P < 0.005; ***P < 0.0005. Data are presented as mean ± SEM, *n* ≥ 9.

To address this, we tested whether oxygenation agent therapy may reprogram the TME and enhance the therapeutic efficacy of adoptively transferred T cells. We utilized a well-established model of murine ACT in which mice bearing 11-day-established pulmonary tumors were infused with 10 × 10^6^ culture-activated tumor-reactive T cells derived from tumor-draining lymph nodes (TDLNs) (69, 70). One hour prior to T cell infusion, mice were treated with Perflubron, and again on days 12, 13, 14, 17, and 19 while housed in the hyperoxic chamber until the study completion on day 21. Figure 5, A and B, and Supplemental Figure 4 demonstrate that oxygenation agent therapy improves the efficacy of ACT since mice treated with this regimen in combination with transferred T cells exhibited significantly decreased numbers of metastatic nodules on the lungs compared with controls. While Perflubron and respiratory hyperoxia alone improved the efficacy of transferred T cells, the combination therapy yielded the strongest tumor regression. Taken together, our data provide evidence that oxygenation agent therapy reprograms the TME via reversal of hypoxia to enhance the antitumor activity of adoptively transferred T cells. These preclinical data may provide justification for testing this strategy in cancer patients that are refractory to current cancer immunotherapies.

### Oxygenation agents in patients with cancer who are unable to receive respiratory hyperoxia.

Our data suggest that the combination of oxygen-carrying Perflubron with respiratory hyperoxia is more effective in inducing tumor regression than either strategy alone. However, there may be clinical situations where the use of respiratory hyperoxia may be limited, especially in patients who would not benefit from systemic exacerbation of the immune system, such as those with lung injuries, ongoing inflammation, or active autoimmunity. In addition, some patients may elect not to remain on respiratory hyperoxia continuously following treatment.

**Figure 6:**
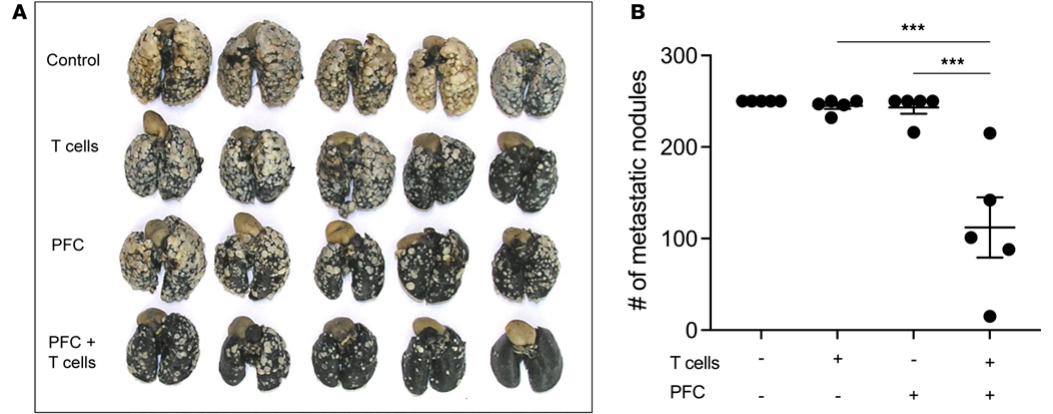
Perflubron administration alone is capable of improving efficacy of adoptive T cell therapy. Mice with 11-day-established pulmonary metastases received ACT of 10 × 10^6^ T cells. One day prior to ACT, mice underwent lymphodepletion with 100 mg/kg i.p. cyclophosphamide. Several hours prior to ACT, mice received i.v. dose of 10 mL/kg of PFC followed by 3 additional doses on days 13, 15, and 17. At the completion of the study (day 19), mice were sacrificed, and metastatic nodules were enumerated by counterstaining with India ink. (**A**) Images of tumor-bearing lungs from each treatment group. (**B**) Quantification of the number of metastatic nodules. *P* values were calculated using 1-way ANOVA with post hoc Tukey’s HSD. ***P < 0.0005. Data are presented as mean ± SEM, *n* = 5.

Therefore, we asked whether Perflubron administered without respiratory hyperoxia may also improve the efficacy of adoptive T cell therapy. Mice with 11-day-established MCA205 fibrosarcoma pulmonary tumors received 10 × 10^6^ T cells derived from TDLNs. One hour prior to ACT, mice were treated with Perflubron, and again on days 13, 15, and 17. On day 19, mice were sacrificed, and pulmonary tumors were enumerated. Figure 6, A and B, and Supplemental Figure 6 demonstrate that Perflubron administration alone can improve the therapeutic efficacy of adoptive T cell therapy since mice receiving Perflubron during ACT demonstrated significantly improved tumor regression compared with control animals. Interestingly, Figure 6A and Supplemental Figure 5 indicate that Perflubron alone (without ACT or respiratory hyperoxia) is able to reduce lung tumor burden, although most lungs had greater than 250 metastases, which is the highest that may be counted reliably. Taken together, data from Figures 5 and 6 suggest that treating patients with oxygenation agent therapy prior to and during ACT can reprogram the TME by reversing hypoxia-adenosinergic suppression and improve therapeutic responses.

## Discussion

These preclinical studies provide evidence to justify the repurposing of available, safe, and well-tolerated blood substitutes to address the medical need in improving existing cancer therapies. ACT using CAR T cells and TCR-transgenic T cells may represent the most powerful immunotherapeutic approach to date since it ensures the presence of sufficient numbers of tumor-reactive immune cells. However, the use of ACT in the clinic has been challenging, particularly in the treatment of solid tumors that are protected by immunological and biochemical negative regulators. While elimination of immunological barriers by PD-1/CTLA4 blockade has yielded improved responses to some cancers, immune cells are still inhibited by the biochemical barriers derived from oxygen-poor and extracellular adenosine–enriched TMEs. Subsequently, the absence of antitumor killer cells in regions of tumor hypoxia weakens the therapeutic efficacy of ICB and ACT, providing justification to target hypoxia to reprogram the TME and improve the success of cancer immunotherapies.

Here, we introduce what we believe is the conceptually novel approach of eliminating the powerful immunosuppressive barrier governed by hypoxia/HIF1A and adenosine → A2AR → cAMP–mediated signaling and immunosuppressive transcription by using oxygenation agents. The presumed novelty of this approach is in utilizing blood substitutes to reprogram the TME by increasing oxygen concentration and weakening the upstream stage of the hypoxia-adenosinergic axis (Figure 1A). The advantage of blood substitutes exists in their substantially smaller size compared with erythrocytes, providing the ability to carry oxygen farther away from the blood vessels and deeper within the disorganized and aberrant tumor microvasculature. This dramatically improves the ability to oxygenate tumors that are in different anatomical locations when compared with more oxygen-privileged organs, such as the lungs (28).

These data provide proof of principle for the use of a class of oxygenation agents (PFCs) to improve cancer immunotherapy by showing that Perflubron (a) carries and delivers oxygen into tumors, (b) eliminates hypoxia in the TME, (c) improves tumor regression and survival, and (d) enhances therapeutic efficacy of adoptive T cell transfer. Additionally, we provide here a methodological approach to increase the amount of dissolved oxygen in PFCs prior to in vivo infusion by saturation with 100% O_2_, and after infusion by exposure to higher fractions of inspired O_2_ achieved by respiratory hyperoxia (60% O_2_).

Finally, our studies further elucidate the relationship between the hypoxic TME and infiltration of tumor-reactive T cells and NK cells. Interestingly, we found a stronger negative correlation between infiltration of NK cells and hypoxic levels when compared with that of CD8^+^ T cells. Our future work aims to clarify the extent to which NK cells may be more sensitive to immunosuppressive hypoxic signaling, which is in accordance with previous studies demonstrating a high susceptibility of NK cells to A2AR-mediated inhibition (28, 29, 65, 66). Indeed, the inhibition of T cells and NK cells by hypoxia/HIF1A- and adenosine-driven transcription is supported by our previous work (24, 25, 27–31, 43). These studies established that hypoxic reversal in the TME using respiratory hyperoxia reduces the transcription of immunosuppressive genes associated with hypoxia response element (HRE) and CRE activity, resulting in a reduction in extracellular adenosine, decreased expression of adenosine receptors, and adenosine-generating ectoenzymes CD39 and CD73, and decreased immunosuppressive molecules (e.g., TGF-β) and reduced suppressor cells (e.g., Tregs) (28, 29). Such observations along with the current understanding of the absence of tumor-reactive T cells and NK cells from hypoxic regions in the TME provide rationale for combining oxygenation agent therapy with cancer immunotherapy protocols.

Data from this study, together with our previous work (7, 27–29), support the following immunotherapeutic protocol where patients with refractory solid tumors may be treated with (a) adoptively transferred T cells (e.g., TCR-transgenic or CAR T cells), (b) a monoclonal antibody (mAb) to inhibit immunological negative regulators, (c) drugs that eliminate extracellular adenosine/A2AR/cAMP–mediated signaling, and (d) oxygenation agents that eliminate or weaken the hypoxia-HIF1A signaling that triggers downstream HRE transcriptional programming and an immunosuppressive adenosine/cAMP axis. In accordance with such a protocol are recent updates from phase I clinical trials (ClinicalTrials.gov NCT03098160) in which 66.7% of patients receiving a combination of evofosfamide (hypoxia-activated prodrug) and ipilimumab achieved stable disease and 16.7% achieved partial response (54). Additionally, patients with preexisting immune gene signatures have been predicted to respond to therapy, which further highlights the necessity of ACT for success of cancer immunotherapies (23, 54, 71, 72).

Our strategy to target tumor hypoxia to improve cancer immunotherapy is also supported by pioneering work by Semenza’s group using pharmacological agents to target HIFs (73, 74). Recently, in a model of murine hepatocellular carcinoma, characterized by high intratumoral hypoxia and poor immunotherapeutic responses, the addition of the HIF inhibitor 32-134D to anti–PD-1 therapy increased the rate of tumor eradication from 25% to 67% (74). Similar to our observations reported here and in our previous work (28, 29), inhibition of HRE-driven transcription led to a reduction in suppressor cells (tumor-associated macrophages and myeloid-derived suppressor cells) and an increase in CD8^+^ T cells and NK cells (74). Our future work will determine whether oxygenation agent therapy may improve responses to ICB.

Importantly, oxygenation via PFCs may benefit from higher fraction of inspired oxygen to maximize O_2_ transport capacity (48, 58, 75). This study identifies a what we believe is a unique treatment modality combining PFC with respiratory hyperoxia (60% O_2_, which previously demonstrated antitumor efficacy by weakening hypoxia-adenosinergic immunosuppression) to improve antitumor immune responses against solid tumors (28, 29). We also show for the first time to our knowledge that an oxygen-delivering nanoemulsion can improve ACT and may also be a powerful method to improve other forms of cancer immunotherapy (43). Extension from this work suggests that treating patients with oxygenation agents prior to ACT may reprogram the TME before arrival of transferred T cells or CAR T cells to improve infiltration, activation, and effector responses. Thus, oxygen delivery via PFCs may offer a safe and effective strategy to target hypoxia-driven immunosuppressive barriers in the TME. Importantly, this treatment option can be readily available, as previous investigation into blood substitutes for several other clinical indications were shown to be safe and well tolerated in over 3,000 patients (58).

### Exclusion criteria in using anti–A2A-adenosinergic drugs to include systemic oxygenation and oxygenation agents.

In this study, we combine PFCs with the accepted medical practice of respiratory hyperoxia (60% O_2_), which is generally considered to be a safe protocol in animals and humans (28, 29, 59, 60). Importantly, here, and in our previous studies with respiratory hyperoxia in tumor models, we did not observe any signs of health concerns, lung dysfunction, or animal morbidity. Supplemental oxygen and PFCs are considered to be much safer than the majority of anticancer drugs, particularly chemotherapeutic agents, but also many other drugs with toxicities and off-target effects. This includes current standard-of-care immunotherapy drugs and clinically approved CAR T cell therapy. Moreover, patients who receive PFCs clinically have received up to 100% FiO_2_ as well as hyperbaric oxygen.

However, it is well established that long-term exposure to oxygen levels much higher than 60% (e.g., 95% O_2_) can cause oxygen toxicity as well as nonspecific inflammatory responses (28, 29, 59, 60). While 60% oxygen is not generally associated with oxygen toxicity, we attract attention here to an important exclusion criterion, in which oxygenation as a therapeutic protocol, since it acts as an anti–A2A-adenosinergic drug, should not be used with cancer patients during episodes of ongoing acute inflammation (60, 76). Respiratory hyperoxia may not only enable stronger antitumor activities by tumor-reactive immune cells, but also stronger inflammatory damage of normal tissues by myeloid cells or T cells activated by other antigens (60, 76).

## Methods

*Sex as a biological variable*. Female mice were used for the tumor immunology assays described since they exhibit more consistent immune responses in these models. Findings are not expected to be relevant to one sex more than the other given the nature of the immunologic pathways that our murine model was designed to investigate. The use of the female sex mice was solely for the purpose of achieving consistency in immunologic responses measured.

*Animals*. Female C57BL/6 or BALB/c wild-type (WT) mice (8–12 weeks old) were purchased from the Jackson Laboratory. Animals were housed in a specific pathogen–free environment in 12-hour/12-hour (light/dark) conditions at approximately 24°C.

*Murine tumor models*. Two different tumor models were employed in these studies: (a) the weakly immunogenic MCA205 fibrosarcoma cell line of B6 origin and (b) the more immunogenic (“hot tumor”) CT26 colon carcinoma purchased from American Type Culture Collection (ATCC). Both cell lines were administered intradermally (i.d.) at doses described in each study and mice were randomized following tumor administration. To provide models of different anatomical location, MCA205 fibrosarcoma was also administered intravenously (i.v.) (0.2 × 10^6^ cells) for experimental pulmonary metastasis. Both cell lines were maintained in complete media containing RPMI-1640 (BioWhittaker) supplemented with 10% heat-inactivated fetal bovine serum (FBS), 100 U/mL penicillin (Gibco), 100 μg/mL streptomycin (Gibco), 0.1 mM nonessential amino acids (Gibco), 1 μM sodium pyruvate (Gibco), 2 mM L-glutamine (Gibco/Thermo Fisher Scientific), 5 × 10^–5^ M 2-mercaptoethanol (Gibco), and 50 μg/mL Gentamicin (Lonza/Fisher Scientific). Cells were cultured at 21% O_2_, 5% CO_2_, and 37°C in Hera Cell Vios 160i incubators (Thermo Fisher Scientific). In vivo inoculation occurred between passages 4 and 6.

### Oxygenation agent therapy

#### Respiratory hyperoxia.

For studies employing respiratory hyperoxia, mice were housed in chambers with well-controlled gas composition to mimic clinical protocols, as done previously (28, 29). Hyperoxia in the chamber is maintained with self-contained oxygen generators (AirSep) to ensure consistent oxygen levels and monitored continuously using oxygen Alpha Omega Instruments oxygen sensors. To avoid hypercapnic acidosis, traditional cage lids were replaced with aerated wire lids and SodaSorb (Medline) was added to absorb any excess carbon dioxide inside the chamber, as done previously (28, 29, 77, 78). Fractional concentrations of O_2_ and CO_2_ inside the chambers were determined previously by pulling a sample from the chamber at a rate of 100 mL/min using a Sable Systems Model SS3 sample pump, as described previously (28, 29).

#### Perflubron preparation for in vivo administration.

Dissolved oxygen is approximately 21% O_2_ in “off-the-shelf” Perflubron since the amount of dissolved O_2_ in PFC-based oxygen carriers is directly proportional to the partial pressure of O_2_ in the surrounding ambient air, which is typically approximately 21% (58) (Figure 3). To maximize oxygen-carrying capacity prior to in vivo administration, Perflubron was infused with 100% O_2_ for 7 minutes and then sealed in an air-tight container with a fitted rubber stopper. To improve injectability, Perflubron was diluted 1:1 in 1× Hank’s Balanced Salt Solution (HyClone, Thermo Fisher Scientific) prior to saturation with 100% O_2_. To minimize reduction of oxygen in the sealed container, Perflubron was withdrawn with a 27-gauge needle for i.v. administration (200 μL) into each mouse and then immediately re-sealed with Parafilm.

### Evaluation of intratumoral hypoxia and the TME

C57BL/6 female mice were injected i.d. with 0.1 × 10^6^ MCA205 fibrosarcoma cells and then randomized. On day 7 after tumor inoculation, mice were placed into the hyperoxic chambers (60% O_2_) for 72 hours or kept at atmospheric oxygen levels (21% O_2_). Tumor-bearing mice receiving oxygenation agent therapy received i.v. Perflubron (15 mL/kg) on days 7, 8, and 9 of tumor growth. One hour before sacrifice, mice were injected intraperitoneally (i.p.) with 80 mg/kg of Hypoxyprobe-1 (pimonidazole HCl) (HP-1000mg, Hypoxyprobe) dissolved in HBSS. Pimonidazole creates thiol protein adducts in tissues with pO_2_ of 10 mmHg or lower. On day 9, mice were sacrificed, and tumors were evaluated for intratumoral hypoxia by immunofluorescent staining of frozen tumor sections and flow cytometry.

#### Immunofluorescence analysis of the TME.

For immunofluorescence analysis, tumors were frozen in Tissue-Tek Optimal Cutting Temperature (OCT) compound (Sakura/Thermo Fisher Scientific) and 5-μm-thick sections were mounted on polypropylene-coated glass slides (Thermo Fisher Scientific) from 10 to 20 different cutting surfaces. Slides were air dried for 45 minutes and fixed in 1:1 mixture of acetone and methanol for 10 minutes at –20°C and stored at –20°C. To detect levels of hypoxia and endogenous immune cell infiltration, slides were first labeled with Fc block (clone 2.4G2, BD Pharmingen) diluted 1:100 in staining buffer (1× PBS, 0.5% bovine serum albumin [BSA], 0.1% Tween 20) for 10 minutes at room temperature in the dark followed by staining with FITC anti-Hypoxyprobe (catalog HP2-1000Kit, Hypoxyprobe), PE anti-CD8 (clone 53-6.7, BioLegend), and APC anti-NK1.1 (clone PK136, BioLegend) mAbs diluted 1:100 for 3 hours. Slides were washed and stained for 5 minutes with 4′,6-diamidino-2-phenylindole (DAPI) (D1306, Thermo Fisher Scientific) for detection of nuclei and mounted with Fluoromount-G (SouthernBiotech). Fluorescently labeled slides were imaged using an IX83 microscope (Olympus) and analyzed using CellSens software (Olympus).

#### Flow cytometric analysis of the TME.

Flow cytometric analysis performed on isolated tumors that were cut into small pieces (2–4 mm) and homogenized using a Murine Tumor Dissociation kit (Miltenyi Biotec) and gentleMACS Dissociator (Miltenyi Biotec) following the manufacturer’s protocol for homogenization of soft/medium tumors. After homogenization, cells were resuspended in 40% Percoll prepared by dilution of 100% Percoll (Cytiva) in culture media prewarmed to room temperature and centrifuged for 10 minutes at 3,000*g*. Lymphocytes and tumor cells were pelleted and debris was separated at the top and removed by aspiration. To remove red blood cells (RBCs), the cell suspension was mixed with 3 mL of ACK Lysing Buffer (Gibco). Remaining cells were stained and acquired using a Cytek DxP 8 flow cytometer and analyzed with FlowJo (BD). Cells were first washed with staining buffer (1× PBS, 1% BSA, 1% penicillin/streptomycin) and centrifuged at 400*g* for 3 minutes to obtain a cell pellet. The supernatant was aspirated, leaving the pelleted cells in approximately 40 μL of staining buffer. Samples were first stained with Fc Block cocktail containing 9.5 μL staining buffer and 0.5 μL Fc Block (clone 2.4G2, BD Pharmingen) for 10 minutes at 4°C. Without washing, 20 μL of surface mAb cocktail was directly added to the cell suspension. Cells were stained for surface markers for 30 minutes at 4°C. For detection of hypoxia, samples were fixed and permeabilized using an eBioscience FoxP3/Transcription Factor Staining Buffer Set (00-5523-00, Invitrogen/Thermo Fisher Scientific) following manufacturer’s protocol. Intracellular staining was performed with FITC anti-Hypoxyprobe mAb for 1 hour at room temperature in permeabilization buffer. Following staining, cells were washed twice and resuspended in staining buffer, passed through a 70-μm filter (Corning), and analyzed on a Cytek DxP 8 flow cytometer. Data were analyzed using FlowJo software.

#### In vivo intratumoral extracellular adenosine measurements.

BALB/c mice with established CT26 intradermal tumors were treated with and without PFC plus 60% O_2_ for 24 hours (*n* = 4 mice per group). Oxygenated Perflubron (15 mL/kg) was given i.v. at 0 and 24 hours, and mice were anesthetized and equilibrium microdialysis probes (Bioanalytical Systems) were inserted into the center of tumors. Microdialysate (isotonic saline, 100 U of heparin) was perfused at 2 mL/min for 2 hours using an infusion pump (Braintree Scientific). Adenosine levels were measured by reversed-phase liquid chromatography–tandem mass spectrometry using a triple quadrupole mass spectrometer with ^13^C_10_-adenosine as an internal standard, as described previously (29).

### Real-time Perflubron-mediated elevation of intratumoral oxygen levels

C57BL/6 female mice were inoculated i.d. with 5 × 10^5^ MCA205 fibrosarcoma cells and randomized. When tumors reached approximately 6 mm × 7 mm, tumor-bearing mice were anesthetized and the tumor was probed using a Presens Oxygen Monitoring System and Oxygen Sensors. After identification of a hypoxic region in the TME (O_2_ < 1%) and stabilization of the readout (~12 minutes), mice received i.v. administration of oxygenated Perflubron (15 mL/kg). Oxygen concentration was recorded for approximately 4 hours (229.5 minutes) after infusion. The experiment was repeated 3 times.

### Tumor growth kinetics and survival studies

Female C57BL/6 mice were inoculated i.d. with 0.1 × 10^6^ MCA205 fibrosarcoma cells resuspended in 100 μL HBSS and randomized. Oxygenation agent therapy was initiated on day 9. For the CT26 colon carcinoma model, 0.75 × 10^5^ tumor cells were injected i.d. into BALB/c WT and oxygenation agent therapy was initiated on day 11. For both in vivo tumor models, oxygenation agent therapy consisted of housing animals in hyperoxic chambers (60% O_2_) and 3 injections/week of 10 mL/kg of Perflubron (i.v.) until study completion. Tumors growth kinetics were measured in a blinded manner 3 times per week using Vernier calipers and volume was calculated using the following formula: π/6 × *H* × *W* × *L*, where *H*, *W*, and *L* are height, width, and length.

### ACT

#### Preparation of culture-activated TDLN T cells for ACT.

Female C57BL/6 donor mice were injected subcutaneously into both flanks with 1 × 10^6^ MCA205 fibrosarcoma cells resuspended in 100 μL HBSS. After 12 days, inguinal TDLNs were isolated and processed to be activated with plate-bound anti-CD3, as described previously (28). One day prior, wells were coated with Protein A from *Staphylococcus* *aureus* (Sigma-Aldrich). After 48 hours, cells were collected and expanded in culture at 0.35 × 10^6^ per mL and supplemented with 100 IU IL-2 for 96 hours in gas-permeable MACS GMP Cell Differentiation Bags (Miltenyi Biotec). After expansion, cells were collected, washed, and resuspended in HBSS. Therapeutic efficacy of transferred T effector cells was assessed in the treatment of 11-day-established MCA205 pulmonary tumors by i.v. injection of 10 × 10^6^ culture-activated T cells into each mouse. Recipient mice were injected i.v. with 2 × 10^5^ MCA205 tumor cells on day 0 for lung tumor establishment. Recipient mice were also pretreated with cyclophosphamide (Sigma-Aldrich; 100 mg/kg i.p.) 1 day before infusion of T cells. Cyclophosphamide treatment is routinely used to improve the therapeutic efficacy of adoptively transferred T cells and was also administered to untreated tumor-bearing control mice (69, 70).

#### ACT and Perflubron in the treatment of pulmonary metastases.

Recipient mice were injected i.v. with 2 × 10^5^ MCA205 tumor cells for lung tumor establishment and randomized. One day prior to adoptive T cell transfer, tumor-bearing recipient mice received lymphodepletion with 100 mg/kg cyclophosphamide. On day 11 of tumor growth, mice received i.v. infusion of 10 × 10^6^ T cells. Several hours prior to ACT, mice were given oxygenated Perflubron i.v. (10 mL/kg). Mice received additional doses of Perflubron (10 mL/kg) on days 13, 15, and 17 until the study completion on day 19. Mice were sacrificed, tumor-bearing lungs were counterstained with India ink, and tumors were enumerated in a blinded manner. Lungs with more than 250 nodules were assigned “>250,” as this is the maximum number that can be counted reliably.

#### ACT and oxygenation agent therapy in the treatment of pulmonary metastases.

Study design was identical to that described above except on day 11 mice were housed in hyperoxic chambers (respiratory hyperoxia [60% O_2_]) or under atmospheric oxygen levels (21% O_2_) until completion of the study on day 21. During this time, mice were treated with or without Perflubron (10 mL/kg) on days 11, 13, 15, 17, and 19. On day 21, mice were sacrificed, tumor-bearing lungs were counterstained with India ink, and tumors were enumerated in a blinded manner. Lungs with more than 250 nodules were assigned “>250,” as this is the maximum number that can be counted reliably.

### Statistics

The significance of differences in the numbers of infiltrating CD8^+^ and NK cells into hypoxic versus nonhypoxic regions, hypoxic exposure of immune cell subtypes, and differences in purine molecules were evaluated using the Student’s *t* test (2-sided). Differences between treatment groups in immunofluorescent staining of frozen tumor sections and flow cytometric assays was evaluated using 1-way ANOVA. For studies of tumor growth kinetics, differences between groups were evaluated using 2-way ANOVA (mixed-effects analysis). All survival studies were analyzed using log-rank test. Significance of differences of lung tumors between groups was evaluated using 1-way ANOVA. All *P* values are listed within the figures or figure legends.

### Study approval

All animal studies were approved by the Institutional Animal Care and Use Committee (IACUC) guidelines of the Division of Laboratory and Animal Medicine at Northeastern University and were conducted according to the NIH *Guide for the Care and Use of Laboratory Animals* (National Academies Press, 2011).

### Data availability

The data generated in this study are provided in the Supporting Data Values file and can also be made available upon request from the corresponding author.

## Author contributions

KHV performed experiments, participated in study design and analysis, and wrote the manuscript together with SMH. SMH led the study from conception to design of experiments, and provided troubleshooting and analysis of data. MVS provided institutional memory of anti–hypoxia-adenosinergic approach and infrastructure at the New England Inflammation and Tissue Protection Institute. MVS also provided constructive criticism and participated in manuscript preparation. BDS provided institutional knowledge of blood substitutes and oxygenation agents, provided Perflubron nanoemulsions used in the studies, and edited the manuscript. MPM, LMW, ACA, NRBR, CB, MS, KMW, and LR assisted in execution of experiments and participated in discussion and analysis of results. VBR and EKJ performed and analyzed all HPLC–tandem mass spectrometry for analysis of the purinome.

## Supplementary Material

Supplemental data

Supporting data values

## Version 1 03/24/2025

Electronic publication
